# Feasibility and preliminary biomarker responses to capsules containing green coffee extract enriched with magnesium di-malate in women with type 2 diabetes *mellitus*: a randomized, placebo-controlled pilot trial

**DOI:** 10.17843/rpmesp.2026.432.15505

**Published:** 2026-06-22

**Authors:** Márcio Flávio Moura de Araújo, José Claudio Garcia Lira Neto, Francisca Sousa Lima Inácio, Thaimara Sousa Freitas Costa, Roberto Wagner Júnior Freire de Freitas, Marta Maria Coelho Damasceno, Maria do Livramento de Paula, Luis Fernando Costa Pereira, Camila Aparecida Pinheiro Landim Almeida, Carla Regina de Souza Teixeira

**Affiliations:** 1 Fundação Oswaldo Cruz (FIOCRUZ), Eusébio, Brasil; 2 Universidade Federal do Piauí, Floriano, Brasil; 3 Universidade da Integração Internacional da Lusofonia Afro-Brasileira (UNILAB), Instituto de Ciências da Saúde, Redenção, Brasil; 4 Universidade Federal do Ceará, Fortaleza, Brasil; 5 Universidade Federal do Maranhão, São Luís, Brasil; 6 Escola de Enfermagem de Ribeirão Preto, Universidade de São Paulo, Ribeirão Preto, Brasil; 7 Universidade Católica Portuguesa, Porto, Portugal

**Keywords:** Coffee, Blood Glucose, Diabetes Mellitus, Type 2, Insulin, Magnesium

## Abstract

**Objetivo.:**

To evaluate the feasibility of a 12-week, randomized, double-blind, placebo-controlled intervention with capsules containing green coffee extract enriched with magnesium di-malate in women with type 2 diabetes *mellitus*, including overall adherence to capsule use and tolerability, as well as to explore preliminary between-group differences in glycemic and cardiovascular biomarkers.

**Materials and methods.:**

This randomized, double-blind, placebo-controlled pilot trial included 48 eligible women with type 2 diabetes *mellitus*, equally assigned to the placebo group (n=24) or the experimental group (n=24). Participants received capsules daily for 12 weeks and were followed up through nursing consultations. Overall retention was 83.3% (40/48), and overall adherence to capsule use was estimated at over 82%. Glycemic biomarkers included fasting glucose, estimated mean glucose, HbA1c, insulin, HOMA-IR, and HOMA-β. Cardiovascular biomarkers included urea, creatinine, uric acid, apolipoprotein B, triglycerides, and the triglycerides-glucose index. Data were analyzed based on available data from participants who completed follow-up and the final evaluation.

**Results.:**

The protocol was feasible under the study conditions, although retention differed between groups. Glycemic biomarkers varied during follow-up in both groups, without conclusive evidence of a consistent between-group effect. In the experimental group, triglycerides and the triglycerides-glucose index showed numerical reductions, while uric acid fluctuated in both groups. These findings should be interpreted as exploratory.

**Conclusions.:**

This pilot trial provided preliminary information on the feasibility of implementing this intervention in women with type 2 diabetes *mellitus*. The biomarker findings were exploratory and imprecise and do not allow for definitive conclusions on efficacy.

## INTRODUCTION

Coffee appears to have the potential to act as a nutraceutical due to its anti-inflammatory, antidiabetic, antiobesity, antioxidant, and hypolipidemic properties, in addition to its relevance as an important food component ^1^. Green coffee, unroasted, is rich in chlorogenic acid and has shown potentially beneficial effects on glucose metabolism and weight control, important aspects in the management of type 2 diabetes mellitus (T2DM) ^2^^-^^4^. However, evidence indicates that clinical findings remain inconsistent regarding the effects of green coffee extract on fasting plasma glucose, insulin, glycated hemoglobin (HbA1c), Homeostatic Model Assessment-Insulin Resistance (HOMA-IR), HDL cholesterol, triglycerides, and liver enzymes ^2^^,^^5^^,^^6^. Another gap identified in previous studies is that none evaluated the effect of this natural product in combination with other substances on glycemic and cardiovascular biomarkers.

In this context, coffee derivatives have been explored as matrices for functional enrichment, including mineral fortification and the incorporation of coffee-derived chlorogenic acids into food systems ^7^^,^^8^. Green coffee is particularly relevant because it constitutes an important source of chlorogenic acids, which have been investigated as bioactive compounds with potential metabolic applications ^3^^,^^7^. In turn, magnesium is biologically relevant for glucose metabolism and insulin action in T2DM ^9^^,^^10^. The most recent evidence from a dose-response meta-analysis of controlled clinical trials also suggests that oral magnesium supplementation can influence glycemic control in patients with T2DM, although the findings remain heterogeneous and dependent on clinical and intervention-related factors ^11^.

Therefore, although no previous clinical studies were identified that specifically evaluated the combination of green coffee extract and magnesium di-malate in women with T2DM, the present intervention was proposed based on the rational foundation of combining a coffee-derived source rich in chlorogenic acids with a mineral involved in glycemic and cardiometabolic regulation. Consequently, this combination should be understood as exploratory and requires a pilot feasibility evaluation before formulating definitive efficacy claims.

Previous reviews suggest that coffee consumption is generally safe in most adults under usual intake conditions, although specific risk groups should be considered ^1^^,^^12^^,^^13^. Therefore, more clinical studies are required to investigate its effects on metabolic and cardiovascular parameters and strengthen the evidence on its use in different populations. Although coffee is widely consumed as an everyday beverage, its translation into a nutraceutical intervention requires careful definition of key parameters, including dose, duration of use, timing of administration, formulation, and combination with other compounds. These aspects are essential for the development of specific, safe, and clinically applicable strategies for different health conditions, including T2DM. From this perspective, a pilot trial represents an important initial step, not to determine definitive efficacy, but to guide the most appropriate direction for the refinement of a future nutraceutical protocol.

Given the inconsistent clinical evidence, the absence of previous studies on this specific combination, and the need to define operational parameters of the intervention, it was considered appropriate to conduct an initial pilot trial. This pilot study aimed to evaluate the feasibility of a 12-week, randomized, double-blind, placebo-controlled intervention with capsules containing green coffee extract enriched with magnesium di-malate in women with T2DM, including adherence to capsule use and tolerability, as well as to explore preliminary effects on glycemic and cardiovascular biomarkers. The study was conducted only in women because middle-aged women with T2DM may experience specific cardiometabolic changes related to the menopausal transition, which can influence glycemic control and cardiovascular risk ^14^^,^^15^. This consideration is especially relevant in studies including glycemic and cardiovascular biomarkers.

**KEY MESSAGES**

Motivation for the study. This pilot trial was conducted to evaluate the feasibility of a 12-week intervention with capsules containing green coffee extract enriched with magnesium di-malate in middle-aged women with type 2 diabetes, in addition to exploring preliminary responses of glycemic and cardiovascular biomarkers.

Main findings. The intervention was feasible under the study conditions, but the findings in biomarkers were exploratory and imprecise and should not be interpreted as definitive evidence of efficacy or inefficacy.

Public health implications. Future definitive trials should incorporate progression criteria, individual adherence monitoring, and follow-up of behavioral variables to improve the interpretation of intervention effects.

## MATERIALS AND METHODS

### Study design

A randomized, double-blind, placebo-controlled, parallel-group pilot trial was conducted, designed to evaluate the feasibility of a 12-week nutraceutical intervention in women with T2DM between November 2023 and March 2024. The study was conceived as a feasibility pilot trial prior to a future definitive randomized clinical trial to evaluate whether recruitment, allocation, follow-up, adherence monitoring, final assessment, and tolerability procedures could be implemented under real clinical conditions.

### Participants and setting

The participants were recruited in the community linked to a primary health care unit in the municipality of Eusebio, Ceara, Brazil. The study dissemination was carried out with the support of community health agents and through messages sent by WhatsApp to women identified from the health unit's patient registry. Recruitment was completed in less than 30 days.

Women with laboratory-confirmed T2DM diagnosis, with more than one year of evolution, aged between 35 and 80 years, and mental capacity to participate in an interview and follow study procedures were eligible. Women diagnosed with thought disorder, severe osteoporosis, chronic glucocorticoid use for more than 45 days, recent surgical intervention in the previous 30 days, pregnancy, or lactation were excluded.

Interested women were initially evaluated by trained nurses, who verified the eligibility criteria, explained the study procedures, and obtained written free and informed consent. A total of 62 women were evaluated to determine their eligibility, and 14 did not meet the eligibility criteria.

**Figure 1:**
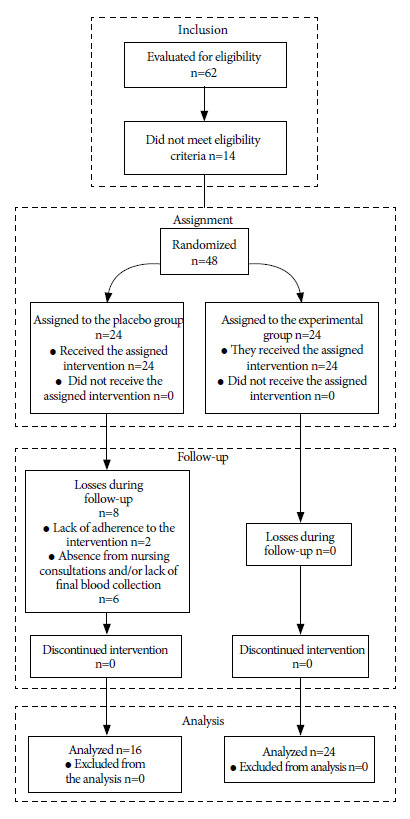
Flowchart for participant selection.

The flow of participants is presented in figure 1.

### Intervention and control

Participants in the experimental group received gelatin capsules containing 500 mg of green coffee extract enriched with 250 mg of di-magnesium malate chelated with malic acid. They were instructed to take one capsule after breakfast and one capsule after lunch, totaling two capsules per day for 12 weeks. The green coffee extract was not analytically quantified regarding the content of chlorogenic acid or caffeine. Participants in the placebo group received gelatin capsules containing 500 mg of carboxymethylcellulose, following the same administration schedule.

The experimental and placebo capsules were prepared by an authorized private compounding pharmacy to produce pharmaceutical formulations in Brazil. The capsules were identical in appearance, color, gelatin coating, packaging, and organoleptic characteristics. They were white capsules, with a gelatin coating, dispensed in identical and sealed bottles. The bottles contained silica packets and cotton to reduce humidity and promote product preservation. The bottles differed only by a discreet numerical code used to identify the assignment.

No dietary changes were promoted during the intervention. The participants continued following the usual care protocols for T2DM from the primary health care unit.

### Feasibility Outcomes

The feasibility of the pilot trial was assessed by recruitment performance, participant retention, completeness of the final biochemical evaluation, adherence to capsule use, and tolerability.

Recruitment yield was defined as the proportion of women screened for eligibility who were finally randomized. Retention was defined as the proportion of randomized participants who completed 12 weeks of follow-up and were included in the final analysis. The completeness of the final biochemical evaluation was defined as the proportion of randomized participants who attended blood sample collection.

Adherence to capsule use was monitored during nursing consultations through self-report and capsule count when participants returned the bottles. Each participant had to consume 168 capsules during the 12-week intervention period, corresponding to two capsules per day for 84 days. When capsule count data were available, individual adherence was calculated using the following formula: Adherence (%) = (number of capsules consumed/168) × 100. However, although participants were instructed at baseline and during follow-up to return the bottles for capsule count, most did not systematically comply with this request. Therefore, individual adherence by capsule count could only be calculated in participants who returned the bottles with complete count data. Overall adherence was estimated based on the total number of capsules administered and consumed according to available records during follow-up, divided by the total number of capsules prescribed.

Tolerability was evaluated through self-reported signs and symptoms during follow-up, including cutaneous, respiratory, and gastrointestinal manifestations. The occurrence of serious adverse events was also monitored during nursing consultations.

### Exploratory biological outcomes

Exploratory biological outcomes included glycemic and cardiovascular biomarkers measured at baseline and at the end of follow-up. Glycemic biomarkers included fasting plasma glucose, estimated mean glucose, glycated hemoglobin, insulin, HOMA-IR, and HOMA-β. Cardiovascular biomarkers included urea, creatinine, uric acid, apolipoprotein B, triglycerides, and triglyceride-glucose index.

Weekly evaluations included capillary glycemia, uric acid, waist circumference, abdominal circumference, and neck circumference. These measurements were used for participant follow-up during the intervention and to explore trends over time.

Detailed information on tolerability based on self-reported signs and symptoms during follow-up, food intake, and biomarker variability is presented in the supplementary material (supplementary material 1, 2, 3, and 4).

### Progression criteria for a future definitive trial

To guide the interpretation of the feasibility and planning of a future definitive randomized clinical trial, progression criteria were established based on the main operational indicators of the pilot study.

Progression to a definitive trial without major modifications was considered feasible if the following criteria were met: recruitment performance ≥70%; overall retention ≥80%; completeness of final biochemical evaluation ≥80%; estimated overall adherence to capsule use ≥75%; and absence of serious adverse events related to the intervention.

The protocol was considered to require modifications before advancing to a definitive trial if global retention or completeness of the final evaluation was between 60% and 79%, if estimated global adherence was between 60% and 74%, or if significant imbalances between groups were observed in retention or symptom occurrence. Values below these thresholds, or the occurrence of serious adverse events related to the intervention, would indicate the need for a substantial revision of the protocol before conducting a definitive trial.

### Sample size

Due to this being a feasibility pilot trial, no formal sample size calculation aimed at efficacy evaluation was performed. The sample size was based on the operational feasibility of recruiting eligible women in the primary health care unit and in the community linked to the service during the study period. In total, 48 eligible women were randomized, with 24 assigned to the experimental group and 24 to the placebo group.

### Randomization, allocation concealment, and blinding

After baseline biochemical results were available, participants were paired according to similar baseline HbA1c values. Within each pair, participants were assigned to one of the two study groups by simple random allocation, resulting in a 1:1 allocation ratio. Randomization was performed by an analyst who had no contact with the participants.

The assignment procedure was performed by a nurse from the research team who had no direct contact with the participants during recruitment or follow-up. Participants were enrolled and accompanied by trained nurses responsible for eligibility assessment, the informed consent process, and weekly consultations. The vials were identified using discrete numerical codes, which allowed distinguishing the assignment group without revealing the intervention content to the participants or the clinical follow-up team.

The study was conducted as a double-blind trial. Participants, follow-up nurses, and outcome assessors remained blinded to group allocation. The experimental and placebo capsules were identical in appearance, color, gelatin coating, packaging, and organoleptic characteristics.

Losses occurring after randomization were considered follow-up losses and did not correspond to losses from the original allocation procedure.

### Data Collection Procedures

Baseline data included sociodemographic characteristics, medical history, medication use, usual coffee consumption, dietary habits, and anthropometric measurements. Blood samples were collected after a 12-hour fast for biochemical analysis. Laboratory analyses were performed by a single contracted laboratory, with the objective of maintaining the consistency of the analytical procedures.

Dietary habits and food consumption frequency were assessed at baseline using a food frequency questionnaire previously validated for the Brazilian adult population. This information was used to characterize the sample and support baseline comparison between groups. Dietary intake, supplement use, habitual coffee consumption outside the intervention, and physical activity were not systematically monitored during follow-up, which was acknowledged as a limitation.

The participants attended weekly nursing consultations during the 12 weeks of intervention. In these consultations, clinical follow-up was performed, the use of the capsules was discussed, signs and symptoms were recorded, and the instruction to return the vials for adherence evaluation was reinforced.

Ten days after concluding the 12-week intervention, the participants returned to the health unit for the final biochemical evaluation, anthropometric measurements, capillary glycemia measurement, and uric acid evaluation.

### Statistical analysis

Data analysis was based on available data from participants who completed follow-up and attended the final evaluation. Feasibility outcomes were summarized using absolute and relative frequencies. Recruitment performance, retention, completeness of the final biochemical evaluation, and adherence indicators were calculated as proportions.

Continuous variables were initially evaluated for normality using the Shapiro-Wilk test. Descriptive statistics were presented using measures of central tendency and variability, according to data distribution. Categorical variables were summarized as frequencies and percentages. Baseline categorical variables, including food consumption categories, were compared between groups using the chi-square test or Fisher's exact test, when appropriate. For dietary habits, "high" and "moderate" consumption categories were grouped into a single category named "non-low," in order to reduce sparse cells and allow for a feasible comparison.

Weekly measurements, including capillary glycemia, uric acid, abdominal circumference, waist circumference, neck circumference, systolic blood pressure, and diastolic blood pressure, were compared between groups using the Mann-Whitney test. Intragroup pre-post comparisons were performed using the Wilcoxon signed-rank test.

Mixed linear models were used to explore changes in biochemical indicators over time according to the study group. The models considered group, time, and the group-time interaction when applicable, with repeated measurements nested within participants. The main assumptions considered for mixed linear models were normality of residuals, homoscedasticity of residuals, independence of observations conditioned on participant-level effects, and appropriate covariance structure for repeated measures.

### Ethical aspects

This study complied with national and international ethical principles for research with human beings. The study was approved by the Research Ethics Committee of the Universidade da Integracao Internacional da Lusofonia Afro-Brasileira, with opinion number 5.865.185/2023. All participants signed the free and informed consent before any study procedure. The trial was registered in the Brazilian Clinical Trials Registry with number RBR-835555x.

## RESULTS

### Feasibility Indicators

Of the 62 women evaluated for eligibility, 48 were considered eligible and randomized, which corresponded to a recruitment yield of 77.4%. Recruitment was completed in less than 30 days through outreach in the community linked to the health service, with the support of community health workers and messages sent via WhatsApp from the unit's patient registry.

Overall retention was 83.3% (40/48). However, a difference was observed between the study arms: retention was 100% in the experimental group (24/24) and 66.7% in the placebo group (16/24). The completeness of the final biochemical evaluation was also 83.3% (40/48). Losses to follow-up occurred only in the placebo group: two participants discontinued the intervention due to non-adherence and six did not attend nursing consultations and/or did not perform the final blood collection.

Individual adherence by capsule count could be calculated in 12 participants who returned the bottles during follow-up. In the other participants, the lack of systematic return of bottles prevented calculating individual adherence by this method. Considering the records available during follow-up, global adherence to capsule use was estimated at over 82%.

No serious adverse events were reported during follow-up. Tolerability was assessed using self-reported signs and symptoms. Overall, symptom frequency was similar between groups, with no evidence of marked differences in tolerability profiles. Abdominal pain and intestinal gas were the most frequently reported complaints during follow-up (supplementary material 1).

### Basal characteristics

The baseline biochemical characteristics of the participants according to study group are presented in table 1. Baseline blood glucose, HbA1c, estimated mean glucose, insulin, HOMA-IR, HOMA-β, TyG index, urea, creatinine, and apolipoprotein B did not show statistically significant differences between the groups. Baseline HbA1c was similar between the groups, with a median of 7.30% in the experimental group and 7.45% in the placebo group. The minimum and maximum HbA1c values in the placebo group were 5.00% and 13.00%, respectively. Baseline triglycerides were higher in the experimental group, with a median of 167.05 mg/dL compared to 102.95 mg/dL in the placebo group (p=0.031). The other baseline biomarkers did not show statistically significant differences between the groups.

**Table 1:** Baseline characteristics of the participants according to study group.

Variable	Experimental group median (min-max)	Placebo group median (min-max)	p value
Fasting blood glucose	119.75 (71.00-467.52)	127.35 (72.61-185.30)	>0.05
HbA1c basal (%)	7.30 (5.03-15.11)	7.45 (5.00-13.00)	>0.05
Estimated Basal Mean Glucose	162.81 (116.89-398.15)	167.18 (122.62-335.01)	>0.05
Basal insulin (microUI/mL)	11.60 (2.50-46.00)	10.15 (5.20-80.71)	>0.05
HOMA-IR basal	3.47 (0.74-21.04)	3.05 (1.48-18.63)	>0.05
HOMA-beta basal	60.4 (2.22-456.00)	62.3 (8.44-949.00)	>0.05
Basal triglycerides (mg/dL)	167.05 (63.41-314.70)	102.95 (52.30-512.10)	0.031
Basal TyG Index	9.38 (7.82-10.60)	8.68 (7.60-10.08)	>0.05
Urea basal (mg/dL)	26.15 (14.12-48.65)	22.25 (11.40-32.50)	>0.05
Basal creatinine (mg/dL)	0.65 (0.50-0.90)	0.60 (0.40-1.00)	>0.05
Basal Apolipoprotein B (mg/dL)	114.00 (66-187)	110.00 (62-151)	>0.05

min: minimum, max: maximum, HbA1c: glycated hemoglobin, HOMA-IR: Homeostatic Model Assessment for Insulin Resistance, HOMA-beta: Homeostatic Model Assessment for beta-cell function, TyG: triglyceride-glucose index.

Group comparisons were performed using the Mann-Whitney test.

The groups were also homogeneous regarding the main health factors at study onset. The predominant skin color was brown in the experimental group (57.1%) and in the placebo group (42.8%) (p=0.821). Most women did not consume alcohol in either group (84.0% and 94.1%, respectively; p=0.551). A significant proportion was hypertensive in both groups (57.0% in the experimental group and 43.0% in the placebo group; p=0.912), and sedentary lifestyle was also frequent (37.0% and 55.6%, respectively; p=0.222). Before the intervention, habitual coffee consumption was up to two cups per day in most participants in the experimental group (63.0%), while in the placebo group, consumption of three to four cups daily predominated in 40.0% of the participants, with no statistically significant difference between the groups (p=0.112).

The sample was predominantly composed of middle-aged women in both groups. The mean number of medications was also similar between the experimental group and the placebo group. Weekly working hours were similar between the groups, with a predominance of the interval greater than 55 weekly hours in both arms of the study.

The detailed information on basal dietary intake patterns is shown in supplementary material 2.

### Exploratory biomarkers during follow-up

During the 12 weeks of follow-up, weekly comparisons between groups for fasting capillary glucose, uric acid, waist circumference, abdominal circumference, and neck circumference did not show a consistent pattern of differences between the groups. In most weeks, p-values were greater than 0.05, indicating no statistically significant differences between the study arms.

In isolation, a statistically significant difference was observed for abdominal circumference at week 9 (p=0.044). The integrated graphical representation of these weekly comparisons is presented in supplementary material 3. In the experimental group, triglycerides and the TyG index showed numerical reductions throughout the follow-up, while uric acid values fluctuated in both groups. However, these variations did not constitute a consistent pattern of difference between the experimental group and the placebo group.

### Mixed Linear Models

The results of the linear mixed models are presented in table 2. No statistically significant differences were identified between the experimental group and the placebo group in the glycemic and cardiovascular biomarkers evaluated. In all cases, the confidence intervals for intergroup differences included the null value, and p-values were greater than 0.05.

**Table 2:** Mixed linear models for glycemic and cardiovascular biomarkers according to study group.

Biomarker	95% CI of the model Lower limit	95% CI of the model Upper limit	95% CI difference between intervention vs. placebo Lower limit	95% CI difference between intervention vs. placebo Upper limit	p-value
Fasting glucose (mg/dL)	81.632	184.132	-78.612	84.405	0.944
Estimated mean glucose (mg/dL)	170.861	259.752	-104.300	364.414	0.344
HbA1c (%)	5.960	9.043	-3.632	1.269	0.344
Insulin (µUI/mL)	3.983	40.549	-17.712	53.113	0.327
HOMA-IR	0.882	9.372	-3.940	9.561	0.414
HOMA-β	0.761	9.273	-3.522	8.331	0.318
Urea (mg/dL)	15.612	34.903	-22.653	8.028	0.350
Creatinine (mg/dL)	0.523	0.794	-0.231	0.198	0.880
Triglycerides (mg/dL)	146.825	253.172	-46.366	122.769	0.375
Apolipoprotein B (mg/dL)	99.598	138.216	-48.552	12.865	0.254
TyG Index	8.865	9.880	-0.502	1.112	0.459

95% CI : 95% confidence interval, HbA1c: glycated hemoglobin, HOMA-IR: Homeostatic Model Assessment for Insulin Resistance, HOMA-β = Homeostatic Model Assessment for β-cell function, TyG: triglyceride-glucose index.

The linear mixed models included group, time, and the group-time interaction, with repeated measurements nested within participants. The estimated difference corresponds to the comparison between the experimental group and the placebo group. The results should be interpreted as exploratory.

### Variability of key biomarkers

The variability of key biomarkers is presented in supplementary material 4. At baseline, HbA1c, triglycerides, and the TyG index showed variability among participants in both groups. Baseline triglycerides were higher in the experimental group, while HbA1c and the TyG index showed more similar initial values between the groups. Dispersion measures, including standard deviation, interquartile range, and coefficient of variation, were incorporated to provide preliminary input for the calculation of the sample size of a future definitive clinical trial.

### Criteria for progression

Based on the established progression criteria, the study reached the defined thresholds for recruitment performance, overall retention, completeness of the final biochemical evaluation, estimated overall adherence, and absence of serious adverse events. Recruitment performance was 77.4% (48/62), overall retention was 83.3% (40/48), and the completeness of the final biochemical evaluation was also 83.3% (40/48). Overall adherence to the use of the capsules was estimated at more than 82%, and no serious adverse events were reported during follow-up.

## DISCUSSION

From a feasibility perspective, this pilot trial showed that the protocol can be implemented in the context of primary care and the community linked to the health service. The study met the predefined progression criteria for recruitment performance, overall retention, completeness of the final biochemical evaluation, estimated overall adherence, and absence of serious adverse events, which suggests that a definitive clinical trial is possible, although it requires operational modifications. However, two aspects indicated the need for modifications before moving toward a definitive trial: the imbalance in retention between groups, with 100% in the experimental group and 66.7% in the placebo group, and the impossibility of calculating individual adherence by capsule count for all participants, due to the low return of bottles during follow-up. Therefore, the protocol was initially considered feasible, but requires adjustments in follow-up, retention, and individual adherence monitoring strategies before moving toward a definitive clinical trial.

These results are consistent with the function of pilot trials, whose purpose is not to demonstrate efficacy, but to verify if the procedures can be sustained before moving towards a definitive trial. Our findings reinforce that this type of intervention can be operationally viable, provided that rigorous strategies for follow-up, adherence control, and monitoring of relevant external variables are established ^16^^-^^18^. The overall retention of 83.3% and the absence of serious adverse events are favorable signs, although the differential loss in the placebo group indicates the need for more robust mechanisms to maintain their participation.

The observed tolerability was similar between the groups. The most frequent discomforts were abdominal pain and intestinal gas. These findings are relevant because coffee-derived products can influence gastrointestinal symptoms, gastric secretion, and digestive irritation in susceptible individuals ^19^^,^^20^. No serious adverse events were observed, which suggests that the capsule-based intervention was generally well tolerated under the evaluated conditions.

Regarding biomarkers, no conclusive effect was observed between the groups on the glycemic and cardiovascular indicators evaluated. The linear mixed models did not identify statistically significant differences between the experimental group and the placebo group, and the confidence intervals of the differences included the null value. Therefore, these results should be interpreted as exploratory and imprecise, consistent with the pilot nature of the study and the limited sample size, and not as definitive evidence of efficacy or inefficacy.

Previous literature suggests that green coffee extract may positively influence glucose metabolism and weight control, especially in studies with animals, healthy volunteers, or overweight or obese individuals, but clinical findings remain heterogeneous ^2^^,^^3^^,^^21^. Likewise, reviews on green coffee extract have shown inconsistent results for fasting glucose, HbA1c, insulin, HOMA-IR, triglycerides, and other cardiometabolic markers ^2^^,^^3^. In this regard, the absence of conclusive differences in our study is compatible with the existing uncertainty in the literature and reinforces the need for trials with a larger sample size, better control of behavioral variables, and greater standardization of the intervention.

The combination of green coffee extract with magnesium di-malate has a plausible biological rationale. The chlorogenic acids from green coffee have been investigated for their possible effects on glucose metabolism, inflammation, and lipid profile ^3^^,^^4^^,^^7^, and magnesium participates in insulin action and glycemic control ^9^^-^^11^. However, the bioavailability of this combination in capsules requires further research. Future studies should also evaluate the form of administration and the standardization of caffeine and chlorogenic acid content, since the food matrix can modify the availability of bioactive compounds ^7^^,^^22^^,^^23^.

The increase in HbA1c observed in both groups must also be interpreted with caution. Although the dose of green coffee extract used was higher than the minimum dose suggested in some studies to influence glycemic parameters ^3^, it cannot be ruled out that unmeasured changes in diet, physical activity, usual coffee consumption, use of supplements, or therapeutic adherence may have influenced glycemic control during follow-up. In this study, these variables were evaluated at baseline, but were not monitored longitudinally. This limitation carries significant weight for the causal interpretation of biomarkers, since unregistered behavioral changes may have contributed to the observed variations in HbA1c and other metabolic indicators.

The sample composition must also be considered in the interpretation of the results. The study exclusively included middle-aged women with T2DM, many of them possibly in climacteric transition or postmenopause. This period is associated with hormonal and cardiometabolic changes that can affect insulin sensitivity, central adiposity, lipid profile, and cardiovascular risk ^10^^,^^12^^,^^24^. Additionally, many participants used antihypertensives, such as hydrochlorothiazide, losartan, and amlodipine, drugs that can influence metabolic and cardiovascular parameters ^25^. These factors may have contributed to the variability of biomarkers and the difficulty in detecting differences between the groups in a pilot sample.

This study presents limitations that should be considered. The sample size was small and the follow-up was relatively short, which limits the precision of the estimates and the ability to detect differences between groups. Likewise, some instruments used for symptom monitoring and operative procedures were reviewed by experts, but did not undergo formal validation with estimation of content validity indices or other psychometric statistics. Although the allocation mechanism was described and the capsules were masked using numerical codes, the allocation and concealment procedure should be refined in a future definitive trial, preferably with centralized random sequence, more robust concealment, and more detailed operational documentation. Another important limitation was the absence of formal acceptability and logistical feasibility metrics. Specific scales or questionnaires were not applied to evaluate participants' perception of the intervention, the ease of use of the capsules, satisfaction with the protocol, or willingness to continue. Likewise, logistical indicators such as nursing team workload, time required for consultations, operational costs, organizational difficulties, or feasibility of larger-scale implementation were not systematically collected. These elements should be incorporated into future pilot or definitive studies to allow a more complete assessment of feasibility. Adherence also represents a relevant limitation. Although overall adherence was estimated at over 82% based on available records, individual adherence by capsule count could not be calculated for all participants due to the low return of bottles. This finding does not invalidate initial feasibility but indicates the need for more rigorous procedures in future trials, such as mandatory bottle return, standardized individual records, phone or electronic reminders, usage diaries, and, if viable, monitoring devices. Similarly, although progression criteria and key biomarker variability measures were incorporated to guide future sample size calculations, these parameters should be prospectively refined in a definitive trial with a larger sample size. The green coffee extract was not analytically quantified in the present study regarding chlorogenic acid or caffeine content. Therefore, the exact content of these compounds in each capsule was not available, which should be considered a limitation related to the standardization and replication of the intervention.

Despite these limitations, the present study provides relevant information on a still little-explored intervention, based on green coffee extract capsules enriched with magnesium di-malate in women with T2DM. More than demonstrating efficacy, the study allowed for the identification of strong and weak points of the protocol, including the speed of recruitment, the feasibility of follow-up, general tolerability, the need to improve placebo group retention, and the importance of longitudinally monitoring behavioral variables. Furthermore, measures of HbA1c, triglyceride, and TyG index variability can contribute to the planning of a future definitive randomized clinical trial.

In conclusion, the intervention with capsules of green coffee extract enriched with magnesium di-malate showed initial feasibility in women with T2DM, meeting the main predefined criteria of the pilot trial. However, the differences in retention between the groups and the limitations in the assessment of individual adherence require adjustments before a definitive trial. Overall, these findings provide relevant information to optimize the design of future larger-scale studies.
