# Supplementary material for: Feasibility and preliminary biomarker responses to capsules containing green coffee extract enriched with magnesium di-malate in women with type 2 diabetes mellitus: a randomized, placebo-controlled pilot trial
Source: Rev Peru Med Exp Salud Publica. 2026 Jun 22;43(2):179–88. doi: 10.17843/rpmesp.2026.432.15505 (PMC13456921; doi:10.17843/rpmesp.2026.432.15505)
Supplement: Supplementary file 2 [file rpmesp-43-02-15505-s001.docx]

**MATERIAL SUPLEMENTARIO**

**Material suplementario 1.** Indicadores de tolerabilidad basados en signos y síntomas autorreportados durante el seguimiento.

| Signo y síntoma | % Sí Grupo experimental | % Sí Grupo placebo | valor p^1^ |
| --- | --- | --- | --- |
| Erupción cutánea | 9.52 | 6.67 | 0.760 |
| Enrojecimiento | 4.76 | - | 0.391 |
| Dificultad respiratoria | 4.76 | - | 0.391 |
| Tos intensa | - | - | - |
| Secreción nasal | 4.76 | 6.67 | 0.890 |
| Lagrimeo excesivo | 19.05 | 6.67 | 0.290 |
| Hipersalivación | 9.52 | - | 0.219 |
| Molestia abdominal | 19.05 | 20.00 | 0.943 |
| Dolor abdominal | 28.57 | 20.00 | 0.558 |
| Vómito | - | - | - |
| Gases intestinales | 28.57 | 20.00 | 0.558 |
| Estreñimiento | 4.76 | - | 0.391 |
| Diarrea | 19.05 | - | 0.073 |

^1^ Comparación entre grupos (prueba de chi-cuadrado)

**Material suplementario 2.** Consumo alimentario de mujeres antes del experimento. Eusébio, Brasil. 2024.

| Categoría de alimentos | Consumo bajo | Consumo no bajo | Consumo bajo | Consumo no bajo | valor p1 |
| --- | --- | --- | --- | --- | --- |
|  | Experimental | | Placebo | |  |
| Carnes y huevos | 15 (57.69%) | 11(42.31%) | 13 (72.22%) | 5 (27.78%) | 0.505 |
| Cereales y leguminosas | 9 (34.62%) | 17 (65.38%) | 7 (38.89%) | 11 (61.11%) | 0.911 |
| Leche y derivados | 17 (65.38%) | 9 (34.62%) | 11 (61.11%) | 7 (38.89%) | 0.477 |
| Snacks y alimentos en conserva | 22 (84.62%) | 4 (15.38%) | 16 (88.89%) | 2 (11.11%) | 0.685 |
| Productos diet y light | 21 (80.77%) | 5 (19.23%) | 11 (61.11%) | 7 (38.89%) | 0.273 |
| Bebidas | 20 (76.92%) | 6 (23.08%) | 15 (83.33%) | 3 (16.67%) | 0.890 |
| Verduras y frutas | 9 (34.62%) | 17 (65.38%) | 5 (27.78%) | 13 (72.22%) | 0.881 |
| Postres y dulces | 26 (100%) | - | 15 (83.33%) | 3 (16.67%) | 0.122 |
| Aceites | 15 (57.69%) | 11 (42.31%) | 14 (77.78%) | 4 (22.22%) | 0.290 |

^1^ Comparación entre grupos (prueba de chi-cuadrado)

**Material suplementario 3.** Evolución semanal de biomarcadores clínicos entre los grupos experimental y placebo durante las 12 semanas de seguimiento. Eusébio, Brasil, 2024.

**Material suplementario 4.** Variabilidad de biomarcadores. Eusébio, Brasil, 2024.

| **Biomarcador** | **Momento** | **Grupo** | **Media ± DE** | **Mediana [RIQ]** | **Mín–máx** | **CV (%)** |
| --- | --- | --- | --- | --- | --- | --- |
| HbA1c (%) | Línea de base | Grupo experimental | 8,28 ± 2,57 | 7,30 [6,70–9,00] | 5,70–15,50 | 31,0 |
| HbA1c (%) | Línea de base | Grupo placebo | 8,09 ± 1,94 | 7,45 [6,85–8,67] | 5,90–13,30 | 24,0 |
| HbA1c (%) | Final | Grupo experimental | 8,34 ± 2,05 | 7,85 [6,92–8,95] | 6,00–13,20 | 24,6 |
| HbA1c (%) | Final | Grupo placebo | 8,68 ± 1,74 | 8,60 [7,50–9,85] | 6,20–13,10 | 20,0 |
| Triglicéridos (mg/dL) | Línea de base | Grupo experimental | 165,33 ± 71,05 | 167,05 [109,52–220,30] | 63,40–314,70 | 43,0 |
| Triglicéridos (mg/dL) | Línea de base | Grupo placebo | 151,24 ± 123,53 | 102,95 [80,05–161,28] | 52,30–512,10 | 81,7 |
| Triglicéridos (mg/dL) | Final | Grupo experimental | 152,02 ± 78,70 | 130,75 [103,93–163,08] | 66,20–404,20 | 51,8 |
| Triglicéridos (mg/dL) | Final | Grupo placebo | 123,44 ± 51,00 | 113,45 [84,80–156,47] | 64,10–235,10 | 41,3 |
| Índice TyG | Línea de base | Grupo experimental | 9,24 ± 0,82 | 9,39 [8,47–9,73] | 7,83–10,61 | 8,9 |
| Índice TyG | Línea de base | Grupo placebo | 8,89 ± 0,71 | 8,68 [8,52–9,42] | 7,60–10,08 | 8,0 |
| Índice TyG | Final | Grupo experimental | 9,01 ± 0,71 | 8,87 [8,44–9,55] | 7,84–10,31 | 7,9 |
| Índice TyG | Final | Grupo placebo | 8,88 ± 0,58 | 8,83 [8,53–9,15] | 8,09–10,21 | 6,5 |

Nota. DE = desviación estándar; RIQ = rango intercuartílico; CV = coeficiente de variación. El CV se presenta para los valores de línea de base y final de cada biomarcador. El índice TyG fue calculado como ln[triglicéridos (mg/dL) × glucosa en ayunas (mg/dL) / 2].
