# Supplementary material for: Feasibility and preliminary biomarker responses to capsules containing green coffee extract enriched with magnesium di-malate in women with type 2 diabetes mellitus: a randomized, placebo-controlled pilot trial
Source: Rev Peru Med Exp Salud Publica. 2026 Jun 22;43(2):179–88. doi: 10.17843/rpmesp.2026.432.15505 (PMC13456921; doi:10.17843/rpmesp.2026.432.15505)
Supplement: Translation

# Translation: Viabilidad y respuestas preliminares de biomarcadores a cápsulas que contienen extracto de café verde enriquecido con di-malato de magnesio en mujeres con diabetes *mellitus* tipo 2: un ensayo piloto aleatorizado y controlado con placebo

**Authors:** Márcio Flávio Moura de Araújo, José Claudio Garcia Lira Neto, Francisca Sousa Lima Inácio, Thaimara Sousa Freitas Costa, Roberto Wagner Júnior Freire de Freitas, Marta Maria Coelho Damasceno, Maria do Livramento de Paula, Luis Fernando Costa Pereira, Camila Aparecida Pinheiro Landim Almeida, Carla Regina de Souza Teixeira

**Affiliations:** 1 Fundação Oswaldo Cruz (FIOCRUZ), Eusébio, Brasil; 2 Universidade Federal do Piauí, Floriano, Brasil; 3 Universidade da Integração Internacional da Lusofonia Afro-Brasileira (UNILAB), Instituto de Ciências da Saúde, Redenção, Brasil; 4 Universidade Federal do Ceará, Fortaleza, Brasil; 5 Universidade Federal do Maranhão, São Luís, Brasil; 6 Escola de Enfermagem de Ribeirão Preto, Universidade de São Paulo, Ribeirão Preto, Brasil; 7 Universidade Católica Portuguesa, Porto, Portugal

**Keywords:** Café, Glucemia, Diabetes Mellitus Tipo 2, Insulina, Magnesio

## Abstract

**Objetivo.:**

Evaluar la factibilidad de una intervención de 12 semanas, aleatorizada, doble ciego y controlada con placebo, con cápsulas que contenían extracto de café verde enriquecido con di-malato de magnesio en mujeres con diabetes *mellitus* tipo 2, incluyendo la adherencia global al uso de las cápsulas y la tolerabilidad, así como explorar diferencias preliminares entre grupos en biomarcadores glucémicos y cardiovasculares.

**Materiales y métodos.:**

Este ensayo piloto aleatorizado, doble ciego y controlado con placebo incluyó a 48 mujeres elegibles con diabetes *mellitus* tipo 2, asignadas equitativamente al grupo placebo (n=24) o al grupo experimental (n=24). Las participantes recibieron cápsulas diariamente durante 12 semanas y fueron seguidas mediante consultas de enfermería. La retención global fue de 83,3% (40/48) y la adherencia global al uso de las cápsulas se estimó en más del 82%. Los biomarcadores glucémicos incluyeron glucosa en ayunas, glucosa media estimada, HbA1c, insulina, HOMA-IR y HOMA-β. Los biomarcadores cardiovasculares incluyeron urea, creatinina, ácido úrico, apolipoproteína B, triglicéridos e índice triglicéridos-glucosa. Los datos se analizaron con base en los datos disponibles de las participantes que completaron el seguimiento y realizaron la evaluación final.

**Resultados.:**

El protocolo fue factible en las condiciones del estudio, aunque la retención difirió entre los grupos. Los biomarcadores glucémicos variaron durante el seguimiento en ambos grupos, sin evidencia concluyente de un efecto consistente entre grupos. En el grupo experimental, los triglicéridos y el índice triglicéridos-glucosa mostraron reducciones numéricas, mientras que el ácido úrico fluctuó en ambos grupos. Estos hallazgos deben interpretarse como exploratorios.

**Conclusiones.:**

Este ensayo piloto aportó información preliminar sobre la factibilidad de implementar esta intervención en mujeres con diabetes *mellitus* tipo 2. Los hallazgos en biomarcadores fueron exploratorios e imprecisos y no permiten establecer conclusiones definitivas sobre eficacia.

## INTRODUCCIÓN

El café parece tener potencial para actuar como nutracéutico debido a sus propiedades antiinflamatorias, antidiabéticas, antiobesidad, antioxidantes e hipolipemiantes, además de su relevancia como importante componente alimentario ^1^. El café verde, sin tostar, es rico en ácido clorogénico y ha mostrado efectos potencialmente beneficiosos sobre el metabolismo de la glucosa y el control del peso, aspectos importantes en el manejo de la diabetes mellitus tipo 2 (DM2) ^2^^-^^4^. No obstante, la evidencia indica que los hallazgos clínicos siguen siendo inconsistentes en relación con los efectos del extracto de café verde sobre la glucosa plasmática en ayunas, la insulina, la hemoglobina glicada (HbA1c), el Homeostatic Model Assessment-Insulin Resistance (HOMA-IR), el colesterol HDL, los triglicéridos y las enzimas hepáticas ^2^^,^^5^^,^^6^. Otra brecha identificada en estudios previos es que ninguno evaluó el efecto de este producto natural en combinación con otras sustancias sobre biomarcadores glucémicos y cardiovasculares.

En este contexto, los derivados del café han sido explorados como matrices para el enriquecimiento funcional, incluyendo la fortificación mineral y la incorporación de ácidos clorogénicos derivados del café en sistemas alimentarios ^7^^,^^8^. El café verde es particularmente relevante porque constituye una fuente importante de ácidos clorogénicos, los cuales han sido investigados como compuestos bioactivos con potenciales aplicaciones metabólicas ^3^^,^^7^. A su vez, el magnesio es biológicamente relevante para el metabolismo de la glucosa y la acción de la insulina en la DM2 ^9^^,^^10^. La evidencia más reciente procedente de un metaanálisis dosis-respuesta de ensayos clínicos controlados también sugiere que la suplementación oral con magnesio puede influir en el control glucémico en pacientes con DM2, aunque los hallazgos siguen siendo heterogéneos y dependientes de factores clínicos y relacionados con la intervención ^11^.

Por lo tanto, aunque no se identificaron estudios clínicos previos que evaluaran específicamente la combinación de extracto de café verde y di-malato de magnesio en mujeres con DM2, la presente intervención se propuso con base en el fundamento racional de combinar una fuente derivada del café rica en ácidos clorogénicos con un mineral implicado en la regulación glucémica y cardiometabólica. En consecuencia, esta combinación debe entenderse como exploratoria y requiere una evaluación piloto de factibilidad antes de formular afirmaciones definitivas de eficacia.

Revisiones previas sugieren que el consumo de café es generalmente seguro en la mayoría de los adultos bajo condiciones habituales de ingesta, aunque deben considerarse grupos de riesgo específicos ^1^^,^^12^^,^^13^. Por lo tanto, se requieren más estudios clínicos para investigar sus efectos sobre parámetros metabólicos y cardiovasculares y fortalecer la evidencia sobre su uso en diferentes poblaciones. Aunque el café se consume ampliamente como bebida cotidiana, su traducción a una intervención nutracéutica requiere una definición cuidadosa de parámetros clave, entre ellos la dosis, la duración del uso, el momento de administración, la formulación y la combinación con otros compuestos. Estos aspectos son esenciales para el desarrollo de estrategias específicas, seguras y clínicamente aplicables para diferentes condiciones de salud, incluida la DM2. Desde esta perspectiva, un ensayo piloto representa un paso inicial importante, no para determinar una eficacia definitiva, sino para orientar la dirección más apropiada para el perfeccionamiento de un futuro protocolo nutracéutico.

Dada la evidencia clínica inconsistente, la ausencia de estudios previos sobre esta combinación específica y la necesidad de definir parámetros operativos de la intervención, se consideró apropiado realizar un ensayo piloto inicial. Este estudio piloto tuvo como objetivo evaluar la factibilidad de una intervención de 12 semanas, aleatorizada, doble ciego y controlada con placebo, con cápsulas que contenían extracto de café verde enriquecido con di-malato de magnesio en mujeres con DM2, incluyendo la adherencia al uso de las cápsulas y la tolerabilidad, así como explorar efectos preliminares sobre biomarcadores glucémicos y cardiovasculares. El estudio se desarrolló solo en mujeres porque las mujeres de mediana edad con DM2 pueden presentar cambios cardiometabólicos específicos relacionados con la transición menopáusica, los cuales pueden influir en el control glucémico y en el riesgo cardiovascular ^14^^,^^15^. Esta consideración es especialmente relevante en estudios que incluyen biomarcadores glucémicos y cardiovasculares.

**MENSAJES CLAVE**

Motivación del estudio. Este ensayo piloto se realizó para evaluar la factibilidad de una intervención de 12 semanas con cápsulas que contenían extracto de café verde enriquecido con di-malato de magnesio en mujeres de mediana edad con diabetes tipo 2, además de explorar respuestas preliminares de biomarcadores glucémicos y cardiovasculares.

Principales hallazgos. La intervención fue factible en las condiciones del estudio, pero los hallazgos en biomarcadores fueron exploratorios e imprecisos y no deben interpretarse como evidencia definitiva de eficacia o ineficacia.

Implicancias en salud pública. Futuros ensayos definitivos deberán incorporar criterios de progresión, monitoreo individual de la adherencia y seguimiento de variables conductuales para mejorar la interpretación de los efectos de la intervención.

## MATERIALES Y MÉTODOS

### Diseño del estudio

Se realizó un ensayo piloto aleatorizado, doble ciego, controlado con placebo y de grupos paralelos, diseñado para evaluar la factibilidad de una intervención nutracéutica de 12 semanas en mujeres con DM2 entre noviembre de 2023 y marzo de 2024. El estudio fue concebido como un ensayo piloto de factibilidad previo a un futuro ensayo clínico aleatorizado definitivo para evaluar si los procedimientos de reclutamiento, asignación, seguimiento, monitoreo de la adherencia, evaluación final y tolerabilidad podían implementarse en condiciones clínicas reales.

### Participantes y entorno

Las participantes fueron reclutadas en la comunidad vinculada a una unidad de atención primaria de salud del municipio de Eusébio, Ceará, Brasil. La divulgación del estudio se realizó con apoyo de agentes comunitarios de salud y mediante mensajes enviados por WhatsApp a mujeres identificadas a partir del registro de pacientes de la unidad de salud. El reclutamiento se completó en menos de 30 días.

Fueron elegibles las mujeres con diagnóstico de DM2 confirmado por laboratorio, con más de un año de evolución, edad entre 35 y 80 años, y capacidad mental para participar en una entrevista y seguir los procedimientos del estudio. Se excluyeron las mujeres con diagnóstico de trastorno del pensamiento, osteoporosis grave, uso crónico de glucocorticoides por más de 45 días, intervención quirúrgica reciente en los 30 días previos, embarazo o lactancia.

Las mujeres interesadas fueron evaluadas inicialmente por enfermeras entrenadas, quienes verificaron los criterios de elegibilidad, explicaron los procedimientos del estudio y obtuvieron el consentimiento libre e informado por escrito. En total, 62 mujeres fueron evaluadas para determinar su elegibilidad y 14 no cumplieron los criterios de elegibilidad.

El flujo de participantes se presenta en la figura 1.

**Figura 1:** Diagrama de flujo de selección de participantes.

### Intervención y control

Las participantes del grupo experimental recibieron cápsulas gelatinosas que contenían 500 mg de extracto de café verde enriquecido con 250 mg de di-malato de magnesio quelado con ácido málico. Fueron orientadas a tomar una cápsula después del desayuno y una cápsula después del almuerzo, totalizando dos cápsulas por día durante 12 semanas. El extracto de café verde no fue cuantificado analíticamente en cuanto al contenido de ácido clorogénico o cafeína. Las participantes del grupo placebo recibieron cápsulas gelatinosas que contenían 500 mg de carboximetilcelulosa, siguiendo el mismo esquema de administración.

Las cápsulas experimentales y las de placebo fueron preparadas por una farmacia privada de manipulación autorizada para elaborar formulaciones farmacéuticas en Brasil. Las cápsulas fueron idénticas en apariencia, color, cubierta gelatinosa, empaque y características organolépticas. Eran cápsulas blancas, con cubierta gelatinosa, dispensadas en frascos idénticos y sellados. Los frascos contenían sobres de sílice y algodón para reducir la humedad y favorecer la conservación del producto. Los frascos diferían únicamente por un código numérico discreto utilizado para identificar la asignación.

No se promovieron cambios dietéticos durante la intervención. Las participantes continuaron siguiendo los protocolos habituales de atención para la DM2 de la unidad de atención primaria de salud.

### Desenlaces de factibilidad

La factibilidad del ensayo piloto se evaluó mediante el rendimiento del reclutamiento, retención de participantes, completitud de la evaluación bioquímica final, adherencia al uso de las cápsulas y tolerabilidad.

El rendimiento del reclutamiento se definió como la proporción de mujeres evaluadas para elegibilidad que fueron finalmente aleatorizadas. La retención se definió como la proporción de participantes aleatorizadas que completaron las 12 semanas de seguimiento y fueron incluidas en el análisis final. La completitud de la evaluación bioquímica final se definió como la proporción de participantes aleatorizadas que asistieron a la extracción de muestras sanguíneas.

La adherencia al uso de las cápsulas fue monitoreada durante las consultas de enfermería mediante autorreporte y conteo de cápsulas cuando las participantes devolvieron los frascos. Cada participante debía consumir 168 cápsulas durante el período de intervención de 12 semanas, correspondiente a dos cápsulas por día durante 84 días. Cuando los datos de conteo de cápsulas estuvieron disponibles, la adherencia individual se calculó mediante la siguiente fórmula: Adherencia (%) = (número de cápsulas consumidas/168) × 100. Sin embargo, aunque las participantes fueron orientadas en la línea de base y durante el seguimiento a devolver los frascos para el conteo de cápsulas, la mayoría no cumplió sistemáticamente esta solicitud. Por ello, la adherencia individual mediante conteo de cápsulas solo pudo calcularse en las participantes que devolvieron los frascos con datos completos de conteo. La adherencia global fue estimada con base en el número total de cápsulas administradas y consumidas según los registros disponibles durante el seguimiento, dividido por el número total de cápsulas prescritas.

La tolerabilidad se evaluó mediante signos y síntomas autorreportados durante el seguimiento, incluyendo manifestaciones cutáneas, respiratorias y gastrointestinales. La ocurrencia de eventos adversos graves también fue monitoreada durante las consultas de enfermería.

### Desenlaces biológicos exploratorios

Los desenlaces biológicos exploratorios incluyeron biomarcadores glucémicos y cardiovasculares medidos en la línea de base y al final del seguimiento. Los biomarcadores glucémicos incluyeron glucosa plasmática en ayunas, glucosa media estimada, hemoglobina glicosilada, insulina, HOMA-IR y HOMA-β. Los biomarcadores cardiovasculares incluyeron urea, creatinina, ácido úrico, apolipoproteína B, triglicéridos e índice triglicéridos-glucosa.

Las evaluaciones semanales incluyeron glucemia capilar, ácido úrico, circunferencia de cintura, circunferencia abdominal y circunferencia de cuello. Estas mediciones se utilizaron para el seguimiento de las participantes durante la intervención y para explorar tendencias a lo largo del tiempo.

La información detallada sobretolerabilidad basados en signos y síntomas autorreportados durante el seguimiento, consumo alimentario y variabilidad de biomarcadores se presenta en el material suplementario (material suplementario 1, 2 ,3 y 4).

### Criterios de progresión para un futuro ensayo definitivo

Para orientar la interpretación de la factibilidad y la planificación de un futuro ensayo clínico aleatorizado definitivo, se establecieron criterios de progresión basados en los principales indicadores operativos del estudio piloto.

La progresión hacia un ensayo definitivo sin modificaciones mayores se consideró factible si se cumplían los siguientes criterios: rendimiento del reclutamiento ≥70%; retención global ≥80%; completitud de la evaluación bioquímica final ≥80%; adherencia global estimada al uso de las cápsulas ≥75%; y ausencia de eventos adversos graves relacionados con la intervención.

Se consideró que el protocolo requeriría modificaciones antes de avanzar hacia un ensayo definitivo si la retención global o la completitud de la evaluación final se situaban entre 60% y 79%, si la adherencia global estimada se situaba entre 60% y 74%, o si se observaban desequilibrios importantes entre los grupos en la retención o en la ocurrencia de síntomas. Valores inferiores a estos umbrales, o la ocurrencia de eventos adversos graves relacionados con la intervención, indicarían la necesidad de una revisión sustancial del protocolo antes de realizar un ensayo definitivo.

### Tamaño de muestra

Debido a que este fue un ensayo piloto de factibilidad, no se realizó un cálculo formal del tamaño de muestra orientado a la evaluación de eficacia. El tamaño de muestra se basó en la factibilidad operativa de reclutar mujeres elegibles en la unidad de atención primaria de salud y en la comunidad vinculada al servicio durante el período del estudio. En total, 48 mujeres elegibles fueron aleatorizadas, con 24 asignadas al grupo experimental y 24 al grupo placebo.

### Aleatorización, ocultamiento de la asignación y enmascaramiento

Después de disponer de los resultados bioquímicos basales, las participantes fueron organizadas en pares según valores similares de HbA1c en la línea de base. Dentro de cada par, las participantes fueron asignadas a uno de los dos grupos del estudio mediante sorteo simple, resultando en una proporción de asignación 1:1. La aleatorización fue realizada por un analista que no tenía contacto con las participantes.

El procedimiento de asignación fue realizado por una enfermera del equipo de investigación que no tuvo contacto directo con las participantes durante el reclutamiento ni durante el seguimiento. Las participantes fueron inscritas y acompañadas por enfermeras entrenadas responsables de la evaluación de elegibilidad, el proceso de consentimiento informado y las consultas semanales. Los frascos fueron identificados mediante códigos numéricos discretos, lo que permitió distinguir el grupo de asignación sin revelar el contenido de la intervención a las participantes ni al equipo clínico de seguimiento.

El estudio fue conducido como un ensayo doble ciego. Las participantes, las enfermeras responsables del seguimiento y los evaluadores de desenlaces permanecieron enmascarados respecto a la asignación de grupo. Las cápsulas experimentales y placebo fueron idénticas en apariencia, color, cubierta gelatinosa, empaque y características organolépticas.

Las pérdidas ocurridas después de la aleatorización fueron consideradas pérdidas de seguimiento y no correspondieron a pérdidas del procedimiento original de asignación.

### Procedimientos de recolección de datos

Los datos basales incluyeron características sociodemográficas, historia clínica, uso de medicamentos, consumo habitual de café, hábitos alimentarios y medidas antropométricas. Las muestras sanguíneas fueron recolectadas después de un ayuno de 12 horas para el análisis bioquímico. Los análisis de laboratorio fueron realizados por un único laboratorio contratado, con el objetivo de mantener la consistencia de los procedimientos analíticos.

Los hábitos dietéticos y la frecuencia de consumo alimentario fueron evaluados en la línea de base mediante un cuestionario de frecuencia alimentaria previamente validado para la población adulta brasileña. Esta información se utilizó para caracterizar la muestra y apoyar la comparación basal entre los grupos. La ingesta dietética, el uso de suplementos, el consumo habitual de café fuera de la intervención y la actividad física no fueron monitoreados sistemáticamente durante el seguimiento, lo que fue reconocido como una limitación.

Las participantes asistieron a consultas semanales de enfermería durante las 12 semanas de intervención. En estas consultas se realizó el seguimiento clínico, se discutió el uso de las cápsulas, se registraron signos y síntomas, y se reforzó la orientación de devolver los frascos para la evaluación de la adherencia.

Diez días después de concluir la intervención de 12 semanas, las participantes regresaron a la unidad de salud para la evaluación bioquímica final, medidas antropométricas, medición de glucemia capilar y evaluación de ácido úrico.

### Análisis estadístico

El análisis de datos se basó en los datos disponibles de las participantes que completaron el seguimiento y asistieron a la evaluación final. Los desenlaces de factibilidad se resumieron mediante frecuencias absolutas y relativas. El rendimiento del reclutamiento, la retención, la completitud de la evaluación bioquímica final y los indicadores de adherencia se calcularon como proporciones.

Las variables continuas fueron evaluadas inicialmente en cuanto a normalidad mediante la prueba de Shapiro-Wilk. Las estadísticas descriptivas se presentaron mediante medidas de tendencia central y variabilidad, de acuerdo con la distribución de los datos. Las variables categóricas se resumieron como frecuencias y porcentajes. Las variables categóricas basales, incluidas las categorías de consumo alimentario, se compararon entre grupos mediante la prueba de chi-cuadrado o la prueba exacta de Fisher, cuando fue apropiado. Para los hábitos alimentarios, las categorías de consumo «alto» y «moderado» se agruparon en una única categoría denominada «no bajo», con el fin de reducir celdas dispersas y permitir una comparación factible.

Las medidas semanales, incluyendo glucemia capilar, ácido úrico, circunferencia abdominal, circunferencia de cintura, circunferencia de cuello, presión arterial sistólica y presión arterial diastólica, se compararon entre grupos mediante la prueba de Mann-Whitney. Las comparaciones intragrupo antes-después se realizaron mediante la prueba de rangos con signo de Wilcoxon.

Se utilizaron modelos lineales mixtos para explorar cambios en los indicadores bioquímicos a lo largo del tiempo según el grupo de estudio. Los modelos consideraron grupo, tiempo y la interacción grupo-tiempo cuando fue aplicable, con mediciones repetidas anidadas dentro de las participantes. Los principales supuestos considerados para los modelos lineales mixtos fueron normalidad de los residuos, homocedasticidad de los residuos, independencia de las observaciones condicionada a los efectos a nivel de participante y estructura de covarianza apropiada para medidas repetidas.

### Aspectos éticos

Este estudio cumplió con los principios éticos nacionales e internacionales para investigaciones con seres humanos. El estudio fue aprobado por el Comité de Ética en Investigación de la Universidade da Integração Internacional da Lusofonia Afro-Brasileira, con dictamen número 5.865.185/2023. Todas las participantes firmaron el consentimiento libre e informado antes de cualquier procedimiento del estudio. El ensayo fue registrado en la Red Brasileña de Ensayos Clínicos con el número RBR-835555x.

## RESULTADOS

### Indicadores de factibilidad

De las 62 mujeres evaluadas para elegibilidad, 48 fueron consideradas elegibles y aleatorizadas, lo que correspondió a un rendimiento de reclutamiento de 77,4%. El reclutamiento se completó en menos de 30 días mediante divulgación en la comunidad vinculada al servicio de salud, con apoyo de agentes comunitarios de salud y mensajes enviados por WhatsApp a partir del registro de pacientes de la unidad.

La retención global fue de 83,3% (40/48). Sin embargo, se observó una diferencia entre los brazos del estudio: la retención fue de 100% en el grupo experimental (24/24) y de 66,7% en el grupo placebo (16/24). La completitud de la evaluación bioquímica final también fue de 83,3% (40/48). Las pérdidas de seguimiento ocurrieron únicamente en el grupo placebo: dos participantes interrumpieron la intervención por no adherencia y seis no asistieron a las consultas de enfermería y/o no realizaron la recolección sanguínea final.

La adherencia individual mediante conteo de cápsulas pudo calcularse en 12 participantes que devolvieron los frascos durante el seguimiento. En las demás participantes, la falta de devolución sistemática de frascos impidió calcular la adherencia individual mediante este método. Considerando los registros disponibles durante el seguimiento, la adherencia global al uso de las cápsulas fue estimada en más de 82%.

No se reportaron eventos adversos graves durante el seguimiento. La tolerabilidad se evaluó mediante signos y síntomas autorreportados. En general, la frecuencia de síntomas fue similar entre los grupos, sin evidencia de diferencias marcadas en los perfiles de tolerabilidad. El dolor abdominal y los gases intestinales fueron las molestias más frecuentemente informadas durante el seguimiento (material suplementario 1).

### Características basales

Las características basales bioquímicas de las participantes según grupo de estudio se presentan en la tabla 1. La glucosa sanguínea basal, la HbA1c, la glucosa media estimada, la insulina, el HOMA-IR, el HOMA-β, el índice TyG, la urea, la creatinina y la apolipoproteína B no mostraron diferencias estadísticamente significativas entre los grupos. La HbA1c basal fue similar entre los grupos, con mediana de 7,30% en el grupo experimental y 7,45% en el grupo placebo. Los valores mínimo y máximo de HbA1c en el grupo placebo fueron 5,00% y 13,00%, respectivamente. Los triglicéridos basales fueron más elevados en el grupo experimental, con mediana de 167,05 mg/dL en comparación con 102,95 mg/dL en el grupo placebo (p=0,031). Los demás biomarcadores basales no presentaron diferencias estadísticamente significativas entre los grupos.

**Tabla 1:** Características basales de las participantes según grupo de estudio.

Variable	Grupo experimental mediana (mín-máx)	Grupo placebo mediana (mín-máx)	Valor de p
Glucosa sanguínea basal	119,75 (71,00-467,52)	127,35 (72,61-185,30)	>0,05
HbA1c basal (%)	7,30 (5,03-15,11)	7,45 (5,00-13,00)	>0,05
Glucosa media estimada basal	162,81 (116,89-398,15)	167,18 (122,62-335,01)	>0,05
Insulina basal (microUI/mL)	11,60 (2,50-46,00)	10,15 (5,20-80,71)	>0,05
HOMA-IR basal	3,47 (0,74-21,04)	3,05 (1,48-18,63)	>0,05
HOMA-beta basal	60,4 (2,22-456,00)	62,3 (8,44-949,00)	>0,05
Triglicéridos basales (mg/dL)	167,05 (63,41-314,70)	102,95 (52,30-512,10)	0,031
Índice TyG basal	9,38 (7,82-10,60)	8,68 (7,60-10,08)	>0,05
Urea basal (mg/dL)	26,15 (14,12-48,65)	22,25 (11,40-32,50)	>0,05
Creatinina basal (mg/dL)	0,65 (0,50-0,90)	0,60 (0,40-1,00)	>0,05
Apolipoproteína B basal (mg/dL)	114,00 (66-187)	110,00 (62-151)	>0,05

mín: mínimo, máx: máximo, HbA1c: hemoglobina glicosilada, HOMA-IR: Homeostatic Model Assessment for Insulin Resistance, HOMA-beta: Homeostatic Model Assessment for beta-cell function, TyG: índice triglicéridos-glucosa.

Las comparaciones entre grupos fueron realizadas mediante la prueba de Mann-Whitney.

Los grupos también fueron homogéneos respecto a los principales factores de salud al inicio del estudio. El color de piel predominante fue pardo en el grupo experimental (57,1%) y en el grupo placebo (42,8%) (p=0,821). La mayoría de las mujeres no consumía alcohol en ninguno de los grupos (84,0% y 94,1%, respectivamente; p=0,551). Una proporción importante era hipertensa en ambos grupos (57,0% en el grupo experimental y 43,0% en el grupo placebo; p=0,912), y el sedentarismo también fue frecuente (37,0% y 55,6%, respectivamente; p=0,222). Antes de la intervención, el consumo habitual de café fue de hasta dos tazas al día en la mayoría de las participantes del grupo experimental (63,0%), mientras que en el grupo placebo predominó el consumo de tres a cuatro tazas diarias en 40,0% de las participantes, sin diferencia estadísticamente significativa entre los grupos (p=0,112).

La muestra estuvo compuesta predominantemente por mujeres de mediana edad en ambos grupos. La cantidad media de medicamentos también fue similar entre el grupo experimental y el grupo placebo. Las horas semanales de trabajo fueron semejantes entre los grupos, con predominio del intervalo superior a 55 horas semanales en ambos brazos del estudio.

La información detallada sobre los patrones de consumo alimentario basal se muestra en el material suplementario 2.

### Biomarcadores exploratorios durante el seguimiento

Durante las 12 semanas de seguimiento, las comparaciones semanales entre los grupos para glucemia capilar en ayunas, ácido úrico, circunferencia de cintura, circunferencia abdominal y circunferencia de cuello no mostraron un patrón consistente de diferencias entre los grupos. En la mayoría de las semanas, los valores de p fueron superiores a 0,05, lo que indica ausencia de diferencias estadísticamente significativas entre los brazos del estudio.

De forma aislada, se observó una diferencia estadísticamente significativa para la circunferencia abdominal en la semana 9 (p=0,044). La representación gráfica integrada de estas comparaciones semanales se presenta en el material suplementario 3. En el grupo experimental, los triglicéridos y el índice TyG mostraron reducciones numéricas a lo largo del seguimiento, mientras que los valores de ácido úrico fluctuaron en ambos grupos. No obstante, estas variaciones no configuraron un patrón consistente de diferencia entre el grupo experimental y el grupo placebo.

### Modelos lineales mixtos

Los resultados de los modelos lineales mixtos se presentan en la tabla 2. No se identificaron diferencias estadísticamente significativas entre el grupo experimental y el grupo placebo en los biomarcadores glucémicos y cardiovasculares evaluados. En todos los casos, los intervalos de confianza de las diferencias entre grupos incluyeron el valor nulo, y los valores de p fueron superiores a 0,05.

**Tabla 2:** Modelos lineales mixtos para biomarcadores glucémicos y cardiovasculares según grupo de estudio.

Biomarcador	IC 95% del modelo Límite inferior	IC 95% del modelo Límite superior	IC 95% diferencia entre intervención vs. placebo Límite inferior	IC 95% diferencia entre intervención vs. placebo Límite superior	Valor de p
Glucosa en ayunas (mg/dL)	81,632	184,132	-78,612	84,405	0,944
Glucosa media estimada (mg/dL)	170,861	259,752	-104,300	364,414	0,344
HbA1c (%)	5,960	9,043	-3,632	1,269	0,344
Insulina (µUI/mL)	3,983	40,549	-17,712	53,113	0,327
HOMA-IR	0,882	9,372	-3,940	9,561	0,414
HOMA-β	0,761	9,273	-3,522	8,331	0,318
Urea (mg/dL)	15,612	34,903	-22,653	8,028	0,350
Creatinina (mg/dL)	0,523	0,794	-0,231	0,198	0,880
Triglicéridos (mg/dL)	146,825	253,172	-46,366	122,769	0,375
Apolipoproteína B (mg/dL)	99,598	138,216	-48,552	12,865	0,254
Índice TyG	8,865	9,880	-0,502	1,112	0,459

IC 95%: intervalo de confianza del 95%, HbA1c: hemoglobina glicosilada, HOMA-IR: Homeostatic Model Assessment for Insulin Resistance, HOMA-β = Homeostatic Model Assessment for β-cell function, TyG: índice triglicéridos-glucosa.

Los modelos lineales mixtos incluyeron grupo, tiempo y la interacción grupo-tiempo, con mediciones repetidas anidadas dentro de las participantes. La diferencia estimada corresponde a la comparación entre el grupo experimental y el grupo placebo. Los resultados deben interpretarse como exploratorios.

### Variabilidad de biomarcadores clave

La variabilidad de los biomarcadores clave se presenta en el material suplementario 4. En la línea de base, la HbA1c, los triglicéridos y el índice TyG mostraron variabilidad entre las participantes de ambos grupos. Los triglicéridos basales fueron más elevados en el grupo experimental, mientras que la HbA1c y el índice TyG presentaron valores iniciales más similares entre los grupos. Las medidas de dispersión, incluyendo desviación estándar, rango intercuartílico y coeficiente de variación, fueron incorporadas para proporcionar insumos preliminares para el cálculo del tamaño muestral de un futuro ensayo clínico definitivo.

### Criterios de progresión

Con base en los criterios de progresión establecidos, el estudio alcanzó los umbrales definidos para rendimiento del reclutamiento, retención global, completitud de la evaluación bioquímica final, adherencia global estimada y ausencia de eventos adversos graves. El rendimiento del reclutamiento fue de 77,4% (48/62), la retención global fue de 83,3% (40/48), y la completitud de la evaluación bioquímica final también fue de 83,3% (40/48). La adherencia global al uso de las cápsulas fue estimada en más de 82%, y no se reportaron eventos adversos graves durante el seguimiento.

## DISCUSIÓN

Desde la perspectiva de la factibilidad, este ensayo piloto mostró que el protocolo puede implementarse en el contexto de la atención primaria y de la comunidad vinculada al servicio de salud. El estudio alcanzó los criterios de progresión definidos para rendimiento del reclutamiento, retención global, completitud de la evaluación bioquímica final, adherencia global estimada y ausencia de eventos adversos graves, lo que sugiere que un ensayo clínico definitivo es posible, aunque requiere modificaciones operativas. Sin embargo, dos aspectos indicaron la necesidad de modificaciones antes de avanzar hacia un ensayo definitivo: el desequilibrio en la retención entre los grupos, con 100% en el grupo experimental y 66,7% en el grupo placebo, y la imposibilidad de calcular la adherencia individual por conteo de cápsulas para todas las participantes, debido a la baja devolución de frascos durante el seguimiento. Por lo tanto, el protocolo fue considerado inicialmente factible, pero requiere ajustes en las estrategias de seguimiento, retención y monitoreo individual de la adherencia antes de avanzar hacia un ensayo clínico definitivo.

Estos resultados son coherentes con la función de los ensayos piloto, cuyo propósito no es demostrar eficacia, sino verificar si los procedimientos pueden sostenerse antes de avanzar hacia un ensayo definitivo. Nuestros hallazgos refuerzan que este tipo de intervención puede ser operacionalmente viable, siempre que se establezcan estrategias rigurosas de seguimiento, control de adherencia y monitoreo de variables externas relevantes ^16^^-^^18^. La retención global de 83,3% y la ausencia de eventos adversos graves son señales favorables, aunque la pérdida diferencial en el grupo placebo indica la necesidad de mecanismos más robustos para mantener su participación.

La tolerabilidad observada fue similar entre los grupos. Las molestias más frecuentes fueron dolor abdominal y gases intestinales. Estos hallazgos son relevantes porque productos derivados del café pueden influir en síntomas gastrointestinales, secreción gástrica e irritación digestiva en individuos susceptibles ^19^^,^^20^. No se observaron eventos adversos graves, lo que sugiere que la intervención basada en cápsulas fue generalmente bien tolerada en las condiciones evaluadas.

En cuanto a los biomarcadores, no se observó un efecto concluyente entre los grupos sobre los indicadores glucémicos y cardiovasculares evaluados. Los modelos lineales mixtos no identificaron diferencias estadísticamente significativas entre el grupo experimental y el grupo placebo, y los intervalos de confianza de las diferencias incluyeron el valor nulo. Por lo tanto, estos resultados deben interpretarse como exploratorios e imprecisos, compatibles con el carácter piloto del estudio y el tamaño muestral limitado, y no como evidencia definitiva de eficacia o ineficacia.

La literatura previa sugiere que el extracto de café verde puede influir positivamente en el metabolismo de la glucosa y en el control del peso, especialmente en estudios con animales, voluntarios sanos o personas con sobrepeso u obesidad, pero los hallazgos clínicos siguen siendo heterogéneos ^2^^,^^3^^,^^21^. Asimismo, revisiones sobre extracto de café verde han mostrado resultados inconsistentes para glucosa en ayunas, HbA1c, insulina, HOMA-IR, triglicéridos y otros marcadores cardiometabólicos ^2^^,^^3^. En este sentido, la ausencia de diferencias concluyentes en nuestro estudio es compatible con la incertidumbre existente en la literatura y refuerza la necesidad de ensayos de mayor tamaño muestral, con mejor control de variables conductuales y mayor estandarización de la intervención.

La combinación de extracto de café verde con di-malato de magnesio tiene un razonamiento biológico plausible. Los ácidos clorogénicos del café verde han sido investigados por sus posibles efectos sobre el metabolismo de la glucosa, la inflamación y el perfil lipídico ^3^^,^^4^^,^^7^, y el magnesio participa en la acción de la insulina y el control glucémico ^9^^-^^11^. Sin embargo, la biodisponibilidad de esta combinación en cápsulas requiere mayor investigación. Estudios futuros deberán evaluar también la forma de administración y la estandarización del contenido de cafeína y ácido clorogénico, ya que la matriz alimentaria puede modificar la disponibilidad de los compuestos bioactivos ^7^^,^^22^^,^^23^.

El aumento de HbA1c observado en ambos grupos también debe interpretarse con cautela. Aunque la dosis de extracto de café verde utilizada fue superior a la dosis mínima sugerida en algunos estudios para influir en parámetros glucémicos ^3^, no se puede descartar que cambios no medidos en dieta, actividad física, consumo habitual de café, uso de suplementos o adherencia terapéutica hayan influido en el control glucémico durante el seguimiento. En este estudio, estas variables fueron evaluadas en la línea de base, pero no fueron monitorizadas longitudinalmente. Esta limitación tiene un peso importante para la interpretación causal de los biomarcadores, ya que cambios conductuales no registrados pueden haber contribuido a las variaciones observadas en HbA1c y otros indicadores metabólicos.

La composición de la muestra también debe considerarse en la interpretación de los resultados. El estudio incluyó exclusivamente mujeres de mediana edad con DM2, muchas de ellas posiblemente en transición climatérica o posmenopausia. Este período se asocia con cambios hormonales y cardiometabólicos que pueden afectar la sensibilidad a la insulina, la adiposidad central, el perfil lipídico y el riesgo cardiovascular ^10^^,^^12^^,^^24^. Además, muchas participantes utilizaban antihipertensivos, como hidroclorotiazida, losartán y amlodipino, fármacos que pueden influir en parámetros metabólicos y cardiovasculares ^25^. Estos factores pueden haber contribuido a la variabilidad de los biomarcadores y a la dificultad para detectar diferencias entre los grupos en una muestra piloto.

Este estudio presenta limitaciones que deben ser consideradas. El tamaño muestral fue reducido y el seguimiento fue relativamente corto, lo que limita la precisión de las estimaciones y la capacidad para detectar diferencias entre grupos. Asimismo, algunos instrumentos utilizados para el seguimiento de síntomas y procedimientos operativos fueron revisados por expertos, pero no pasaron por una validación formal con estimación de índices de validez de contenido u otros estadísticos psicométricos. Aunque el mecanismo de asignación fue descrito y las cápsulas fueron enmascaradas mediante códigos numéricos, el procedimiento de asignación y ocultamiento debe ser perfeccionado en un futuro ensayo definitivo, preferiblemente con secuencia aleatoria centralizada, ocultamiento más robusto y documentación operativa más detallada. Otra limitación importante fue la ausencia de métricas formales de aceptabilidad y de viabilidad logística. No se aplicaron escalas o cuestionarios específicos para evaluar la percepción de las participantes sobre la intervención, la facilidad de uso de las cápsulas, la satisfacción con el protocolo o la disposición a continuar. Asimismo, no se recogieron de forma sistemática indicadores logísticos como carga de trabajo del equipo de enfermería, tiempo requerido para las consultas, costos operativos, dificultades organizativas o factibilidad de implementación a mayor escala. Estos elementos deberán incorporarse en futuros estudios piloto o definitivos para permitir una evaluación más completa de la factibilidad. La adherencia también representa una limitación relevante. Aunque la adherencia global fue estimada en más de 82% con base en los registros disponibles, la adherencia individual por conteo de cápsulas no pudo calcularse para todas las participantes debido a la baja devolución de frascos. Este hallazgo no invalida la factibilidad inicial, pero indica la necesidad de procedimientos más rigurosos en futuros ensayos, como devolución obligatoria de frascos, registros individuales estandarizados, recordatorios telefónicos o electrónicos, diarios de uso y, si es viable, dispositivos de monitoreo. Del mismo modo, aunque se incorporaron criterios de progresión y medidas de variabilidad de biomarcadores clave para orientar futuros cálculos muestrales, estos parámetros deberán ser refinados prospectivamente en un ensayo definitivo con mayor tamaño muestral. El extracto de café verde no fue cuantificado analíticamente en el presente estudio en cuanto al contenido de ácido clorogénico o cafeína. Por lo tanto, el contenido exacto de estos compuestos en cada cápsula no estuvo disponible, lo que debe considerarse una limitación relacionada con la estandarización y la replicación de la intervención.

A pesar de estas limitaciones, el presente estudio aporta información relevante sobre una intervención todavía poco explorada, basada en cápsulas de extracto de café verde enriquecido con di-malato de magnesio en mujeres con DM2. Más que demostrar eficacia, el estudio permitió identificar puntos fuertes y débiles del protocolo, incluyendo la rapidez del reclutamiento, la factibilidad del seguimiento, la tolerabilidad general, la necesidad de mejorar la retención del grupo placebo y la importancia de monitorear longitudinalmente variables conductuales. Además, las medidas de variabilidad de HbA1c, triglicéridos e índice TyG pueden contribuir a la planificación de un futuro ensayo clínico aleatorizado definitivo.

En conclusión, la intervención con cápsulas de extracto de café verde enriquecido con di-malato de magnesio mostró factibilidad inicial en mujeres con DM2, cumpliendo los principales criterios predefinidos del ensayo piloto. Sin embargo, las diferencias en la retención entre los grupos y las limitaciones en la evaluación de la adherencia individual requieren ajustes antes de un ensayo definitivo. En conjunto, estos hallazgos aportan información relevante para optimizar el diseño de futuros estudios de mayor escala.
